# Integrated microarray and multiplex cytokine analyses of Kaposi's Sarcoma Associated Herpesvirus viral FLICE Inhibitory Protein K13 affected genes and cytokines in human blood vascular endothelial cells

**DOI:** 10.1186/1755-8794-2-50

**Published:** 2009-08-06

**Authors:** Vasu Punj, Hittu Matta, Sandra Schamus, Preet M Chaudhary

**Affiliations:** 1 Department of Medicine, Division of Hematology-Oncology, Hillman Cancer Center, University of Pittsburgh Cancer Institute, University of Pittsburgh, Pittsburgh, PA, USA

## Abstract

**Background:**

Kaposi's sarcoma (KS) associated herpesvirus (KSHV) is the etiological agent of KS, a neoplasm characterized by proliferating spindle cells, extensive neoangiogenesis and a prominent inflammatory infiltrate. Infection of blood vascular endothelial cells with KSHV in vitro results in their spindle cell transformation, which is accompanied by increased expression of inflammatory chemokines and cytokines, and acquisition of lymphatic endothelial markers. Mimicking the effect of viral infection, ectopic expression of KSHV-encoded latent protein vFLIP K13 is sufficient to induce spindle transformation of vascular endothelial cells. However, the effect of K13 expression on global gene expression and induction of lymphatic endothelial markers in vascular endothelial cells has not been studied.

**Methods:**

We used gene array analysis to determine change in global gene expression induced by K13 in human vascular endothelial cells (HUVECs). Results of microarray analysis were validated by quantitative RT-PCR, immunoblotting and a multiplex cytokine array.

**Results:**

K13 affected the expression of several genes whose expression is known to be modulated by KSHV infection, including genes involved in immune and inflammatory responses, anti-apoptosis, stress response, and angiogenesis. The NF-κB pathway was the major signaling pathway affected by K13 expression, and genetic and pharmacological inhibitors of this pathway effectively blocked K13-induced transcriptional activation of the promoter of CXCL10, one of the chemokines whose expression was highly upregulated by K13. However, K13, failed to induce expression of lymphatic markers in blood vascular endothelial cells.

**Conclusion:**

While K13 may account for change in the expression of a majority of genes observed following KSHV infection, it is not sufficient for inducing lymphatic reprogramming of blood vascular endothelial cells.

## Background

Infection with Kaposi's Sarcoma (KS)-associated herpesvirus (KSHV), also known as the Human herpesvirus 8 (HHV8), has been linked to the development of Kaposi's sarcoma (KS), primary effusion lymphoma and multicentric Castleman's disease [1] KS is a highly vascular tumor that is induced by the infection of vascular or lymphatic endothelial cells with KSHV and is characterized by the presence of distinctive proliferating spindle-like cells, prominent neoangiogenesis and infiltration by inflammatory cells [2,3]. The spindle cells not only represent the tumor cells in the KS lesion, but also produce a number of proinflammatory and angiogenic factors that drive the growth of the lesion [3]. Latent infection of both micro- and macro-vascular endothelial cells with KSHV *in vitro *makes them acquire a spindle cell phenotype, which is accompanied by increased expression of a number of genes involved in the regulation of immune and inflammatory responses, cellular stress, apoptosis and angiogenesis [4-6]. Interestingly, KSHV infection of blood vascular endothelial cells also upregulates the expression of several of lymphatic markers, such as PROX-1, VEGFR-1, Podoplanin and XLKD1/LYVE1, which has led to the suggestion that KSHV infection results in lymphatic reprogramming of vascular endothelial cells [7-9].

The KSHV-encoded K13 protein is one of the few proteins to be expressed in latently-infected spindle cells. Although originally classified as a viral FLICE inhibitory protein (vFLIP), K13 was subsequently shown to be a potent activator of the NF-κB pathway [10-12], and to use this pathway to promote cellular survival, proliferation, transformation, cytokine secretion and KSHV latency [13-20]. Ectopic expression of K13 in human vascular endothelial cells is sufficient to transform them into spindle cells, which is accompanied by the upregulated expression of several proinflammatory cytokines and adhesion molecules known to be induced in KSHV-infected vascular endothelial cells [21,22]. However, the effect of K13 on global gene expression in vascular endothelial cells has not been studied. It is also not clear whether ectopic expression of K13 in vascular endothelial cells, in the absence of other KSHV latent genes, is sufficient for inducing the changes in gene expression observed following infection with KSHV. To address these questions, we have examined the effect of ectopic K13 expression on global gene expression in human vascular endothelial cells (HUVECs). Our results indicate that K13 may account for change in the expression of a significant proportion of genes observed following KSHV infection. However, in contrast to KSHV infection, ectopic expression of K13 is incapable of inducing the expression of lymphatic endothelial markers.

## Methods

### Cells used in this study

Human Umbilical Vein Endothelial Cells (HUVECs) were purchased from Cambrex (East Rutherford, NJ) and were grown in EMB medium containing 10% FBS (fetal bovine serum) and supplemented with the bullet kit. Cells were used for experiments at passages 2 to 6. HUVECs stably transduced with an MSCVneo vector expressing a 4-Hydroxytamoxifen (4OHT)-inducible K13-ER^TAM ^construct were selected in G418 and have been described previously [21]. These cells were maintained under G418 selection for several passages prior to being used in the experiments to ensure that the experiments were conducted with stably transduced cells. An independent population of HUVECs stably transduced with a MSCV-hygro vector encoding the K13-ER^TAM ^fusion construct were also generated and used to confirm the results of the microarray analysis.

### Gene chip human array

We used the human genome HGU-133 plus 2.0 arrays (Affymatrix, Santa Clara, CA), an oligonucleotide-probe based gene array chip containing ~50,000 transcripts, which provides a comprehensive coverage of the whole human genome.

### RNA isolation and hybridization to oligonucleotide arrays

HUVECs stably expressing empty vectors (MSCVneo and MSCV-hygro) or K13-ER^TAM^-encoding constructs were treated with 4OHT (50 nM) or solvent for 48 h. Total RNA was isolated using Qiagen RNeasy kit (Qiagen, Valencia, CA). Ten micrograms of total RNA was used to synthesize cDNA. T7 promoter introduced during the first strand synthesis was then used to direct cRNA synthesis, which was labeled with biotinylated deoxynucleotide triphosphate, following the manufacturer's protocol (Affymatrix, San Diego, California). After fragmentation, the biotinylated cRNA was hybridized to the gene chip array at 45°C for 16 h. The chip was washed, stained with phycoerytherin-streptavidin, and scanned with the Gene Chip Scanner 3000. After background correction, preliminary data analysis was done in the Microarray Suite 5.0 software (MAS 5.0, Stratagene, La Jolla, CA). For primary analysis we used PLIER as recommended in the work flow of software Gene Spring GX10.0 (Agilent Technologies, Santa. Clara).

### Gene array data analysis

Fluorescence intensities were uploaded to the Array Assist 6.5 and Gene Spring GX10.0 (Agilent Technologies, Santa Clara) software. Data was normalized by quantitative normalization, and then transferred logarithmically for further analysis to determine changes in a particular gene induced by K13. In order to compare the changes in gene expression, the data was further normalized by using the 50 RFU fluorescence value as threshold, and statistical analysis showing fold changes was determined (*p *≤ 0.05). The microarray experiment design, setup, and data have been deposited in National Center for Biotechnology Information's Gene Expression Omnibus and are accessible through GEO series accession number GSE16051.

### Luciferase reporter assay

A luciferase reporter plasmid containing the CXCL10/IP-10 promoter (pGL3IP-10) was kindly provided by Dr. Dan Muruve (University of Calgary). Expression constructs for K13, K13-58AAA and MC159 have been described previously [14]. 293T cells were transfected in a 24-well plate with various test plasmids along with the CXCL10 luciferase reporter constructs (50 ng/well) and a pRSV/LacZ (β-galactosidase) reporter construct (75 ng/well), as described previously [10]. Cells were lysed 24-36 h later, and cell extracts were used to measure firefly luciferase and β-galactosidase activities, respectively. Luciferase activity was normalized by the β-galactosidase activity to control for differences in transfection efficiency.

### Western blot

Western blot analysis was performed as described previously [21]. Primary antibody dilutions used in these experiments were Flag (1:5000; Sigma, St. Louis), COX2 (1:1000; Cayman Chemicals, Michigan), β-actin (1:5000; Sigma, St. Louis), IκB-α (1:2000: Santa Cruz, Santa Cruz, CA) and Tubulin ((1:1000; Sigma, St. Louis).

### Network, gene ontology and canonical pathways analysis

Genes, which qualified in the stringent statistical tests, were used for gene ontology and pathway analysis. Expression data sets containing gene identifier and their corresponding expression values, as fold-changes, were uploaded as a tab-delimited text file to the Ingenuity pathway Analysis (IPA) software (Ingenuity systems, Mountain view, CA). Genes, which mapped to the ingenuity pathway database, were categorized based on molecular functions, gene ontology and biological processes. Each class was grouped based on their p-value. The identified genes named as focused genes were also mapped to genetic networks in the IPA database and ranked by score. The calculated probability score represented whether a collection of genes in a network could be found by chance alone.

### mRNA expression assay by quantitative reverse transcript-polymerase chain reaction (qRT-PCR)

cDNA was synthesized from RNA samples by PCR RNA core kit (Applied Biosystems, Bedford, MA). Real time quantitative reverse transcript-polymerase chain reaction (qRT-PCR) with SYBER Green, using gene-specific PCR primers, was performed to verify the microarray data. Eleven genes were selected randomly and the primers used to amplify each gene are listed in the Additional File-1. Samples were run in triplicate, and PCR was performed by an ABI 7700 thermocycler (Applied Biosystems, Bedford, MA). Expression of multiple house keeping genes (GNB, β-actin, GAPDH and tubulin) were simultaneously determined for normalization, following the geNorm method [23]. A linear regression analysis was performed and the coefficient of variation was calculated to assess a correlation between the RT-PCR and gene array results of these 11 randomly selected genes.

### Multiplex analysis for cytokines and chemokines affected by K13

The LabMAP technology (Luminex) combines the principle of a sandwich immunoassay with the fluorescent-bead-based technology. A Luminex-based Multiplexed assay was purchased from Biosource International (Camarillo, CA) and used to measure the presence of cytokines and chemokines in culture supernatants of HUVEC-K13-ER^TAM ^that had been mock-treated or treated with 4OHT for 48 h. For comparison, supernatant from HUVECs infected with KSHV for 48 h was included.

## Results

### Induction of host gene expression by K13

In our previous work, we used retroviral-mediated gene transfer to generate human umbilical endothelial cells (HUVECs) with stable expression of a K13-ER^TAM ^fusion construct in which the K13 cDNA is fused in-frame to the ligand-binding domain of a mutated estrogen receptor [21]. The mutated estrogen receptor does not bind to its physiological ligand estrogen, but binds with very high affinity to the synthetic ligand 4OHT (4-hydroxytamoxifen) and regulates the activity of K13 in a 4OHT-dependent manner [21]. In the absence of 4OHT treatment, the K13-ER^TAM^-HUVECs maintain their cobblestone appearance and are indistinguishable from the empty vector-expressing cells in their growth characteristics. However, these cells acquire spindle morphology within 24 h of induction by 4OHT treatment, thus mimicking the effect of KSHV infection [21].

To comprehensively identify the spectrum of genes induced by K13 expression, HUVECs with long-term stable expression of empty vector (MSCV) and K13-ER^TAM ^were plated in parallel cultures. Each group of cells was then mock treated or treated with 4OHT for 48 h and RNA was harvested from all plates simultaneously. The RNA was then quantified and subjected to high density oligonucleotide microarray analysis using the Affymetrix HG-U133 plus 2 gene array representing ~50,000 annotated transcripts.

**Figure 1:**
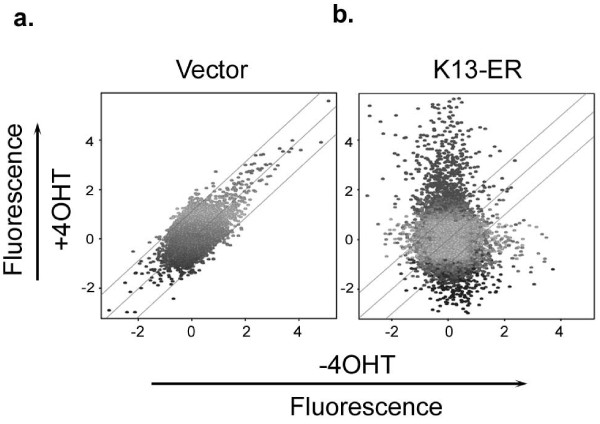
**(a & b). Scatter plot analysis of genes induced by 4OHT treatment in the control vector and K13-ER^TAM^-expressing HUVECs**. Each point on the scatter plots represents the expression of an individual mRNA message, as determined by units of fluorescent intensity, in untreated cells (*x *axis) plotted against its expression after 4OHT treatment (*y *axis). The lines on scatter plot indicate the 2-fold boundaries used for selecting genes with differential expression. The 4OHT-regulated genes fall outside of these lines. Only one of the two experiments is presented; similar results were obtained with the duplicate experiment.

We used several well established data mining tools to analyze the microarray data. The change in gene expression, expressed as fluorescence, was first examined by scatter plot analysis. In the control vector (MSCV)-expressing HUVECs, the fluorescence ratios of most of the genes with and without 4OHT treatment remained close to 1 or changed slightly (≤2), suggesting that 4OHT by itself did not have significant effect on gene expression (Figure 1a). In contrast, in the K13-ER^TAM^-expressing HUVECs, the fluorescence ratios of a significant proportion of genes was changed more than 2 fold following 4OHT treatment (Figure 1b).

**Figure 2:**
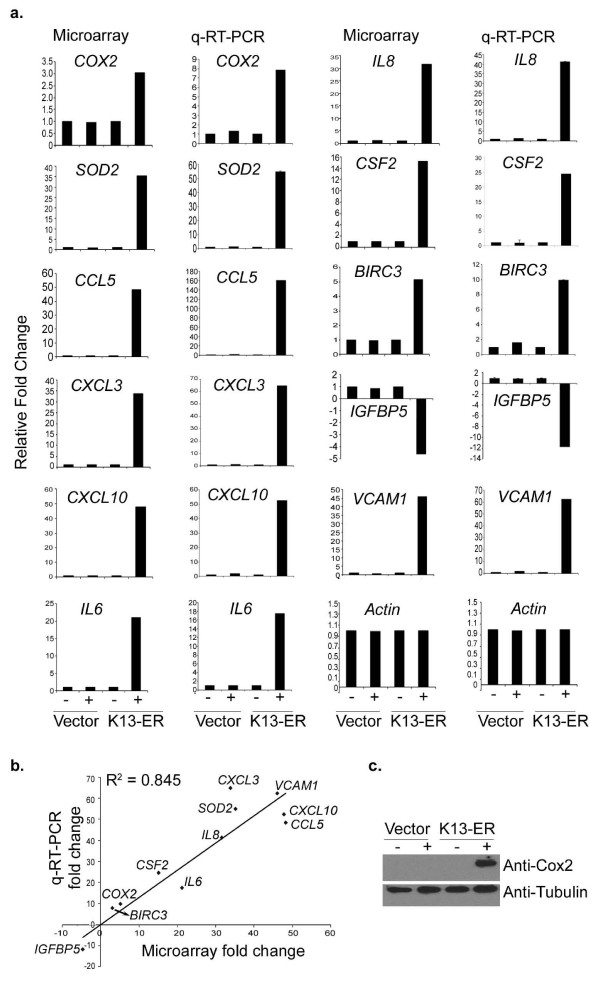
**Validation of gene expression array data by qRT-PCR and western blotting**. **(a)**. Eleven genes were randomly picked and their relative mRNA levels in the mock- and 4OHT- treated vector and K13-ER^TAM ^expressing HUVECs were examined using real-time RT-PCR. The two different methods, the microarrays and the RT-PCR, show excellent qualitative agreement. **(b)**. Linear regression analysis shows excellent correlation between the qRT-PCR and microarray data with a coefficient of variation (R^2^) of 0.845. **(c)**. Western blot analysis confirms induction of COX-2/PTGS2 upon treatment of K13-ER^TAM ^cells with 4OHT. Tubulin blot shows equal protein loading.

In order to validate our gene array data, 11 genes were randomly chosen (COX2/PTGS2, CSF2, CXCL10, CXCL3, hIL8, hIL6, IGFB5, RANTES/CCL5, SOD2, and VCAM-1) and their expression was measured by real time RT-PCR (qRT-PCR). This group included both up- and down-regulated genes. To our satisfaction, there was a good correlation between the qRT-PCR and gene array data (R^2 ^= 0.845) (Figure 2a and 2b). To correlate the gene array and qRT-PCR detected transcription changes with protein levels, we analyzed the expression levels of COX2/PTGS2 protein by immunoblot and confirmed its strong induction in K13-ER^TAM^-expressing HUVECs following 4OHT treatment (Figure 2c). Additional validation of microarray data at the protein level was obtained by a multiplex cytokine assay (see below). Taken collectively, there was a good correlation between array and other means of target verification, thereby validating our methodology and analysis.

### Identification of genes differentially affected by K13

In order to identify genes that were differentially affected by 4OHT treatment in the HUVEC-K13-ER^TAM^, the gene array data was uploaded to Array Assist^® ^gene expression software. It was mean shift normalized and primary analysis was performed using PLIER, as recommended in the workflow of the software. Only 174 genes, which showed more than two fold difference in 4OHT-treated over mock-treated cells (*p *< 0.01), met the stringency of this statistical analysis. Of these 174 genes, 123 genes were upregulated, whereas 51 genes were down-regulated. A list of 50 most upregulated and 15 most down-regulated genes are provided in Table 1 and a complete list of all K13 affected genes is provided in Additional File 2. A similar effect of K13 induction on global gene expression was observed in the HUVECs stably transduced with the MSCV hygro-K13-ER^TAM ^construct (data not shown).

**Table 1:** List of most differentially regulated genes in 4OHT-treated K13 ER-HUVECs.

	**Gene Name**	**Gene**	**Entrez Gene**	**Fold Change**
**Up-regulated genes**				

1	*chemokine (C-C motif) ligand 2*	**CCL2**	6347	71.4
2	*ubiquitin D*	**UBD**	10537	58.4
3	*chemokine (C-C motif) ligand 20*	**CCL20**	6364	53.1
4	*chemokine (C-C motif) ligand 5*	**CCL5**	6352	48.3
5	*chemokine (C-X-C motif) ligand 10*	**CXCL10**	3627	47.7
6	*vascular cell adhesion molecule 1*	**VCAM1**	7412	46.0
7	*selectin E (endothelial adhesion molecule 1)*	**SELE**	6401	35.4
8	*superoxide dismutase 2, mitochondrial*	**SOD2**	6648	35.2
9	*chemokine (C-X-C motif) ligand 3*	**CXCL3**	2921	33.8
10	*chemokine (C-X-C motif) ligand 2*	**CXCL2**	2920	32.2
11	*interleukin 8*	**IL8**	3576	31.5
12	*chromosome 15 open reading frame 48*	**C15orf48**	84419	29.6
13	*solute carrier family 7 (cationic amino acid transporter)*	**SLC7A2**	6542	23.2
14	*chemokine (C-X3-C motif) ligand 1*	**CX3CL1**	6376	22.3
15	*intercellular adhesion molecule 1 (CD54)*	**ICAM1**	3383	22.2
16	*tumor necrosis factor, alpha-induced protein 6*	**TNFAIP6**	7130	21.2
17	*interleukin 6 (interferon, beta 2)*	**IL6**	3569	21.1
18	*colony stimulating factor 2 (granulocyte-macrophage)*	**CSF2**	1437	15.1
19	*interferon stimulated exonuclease gene 20 kDa*	**ISG20**	3669	13.6
20	*chemokine (C-X-C motif) ligand 5*	**CXCL5**	6374	13.0
21	*laminin, gamma 2*	**LAMC2**	3918	12.7
22	*tumor necrosis factor, alpha-induced protein 3*	**TNFAIP3**	7128	12.5
23	*matrix metallopeptidase 10 (stromelysin 2)*	**MMP10**	4319	11.5
24	*tumor necrosis factor, alpha-induced protein 2*	**TNFAIP2**	7127	11.3
25	*Epstein-Barr virus induced gene 3*	**EBI3**	10148	9.8
26	*tumor necrosis factor (ligand) superfamily, member 13b*	**TNFSF13B**	10673	9.6
27	*phospholipase A1 member A*	**PLA1A**	51365	9.4
28	*GTP cyclohydrolase 1 (dopa-responsive dystonia)*	**GCH1**	2643	9.0
29	*nuclear factor of kappa light polypeptide gene enhancer, alpha*	**NFKBIA**	4792	8.3
30	*proteasome (prosome, macropain) subunit, beta type, 9*	**PSMB9**	5698	7.9
31	*Chemokine (C-X-C motif) 1*	**CXCl1**	2919	7.7
32	*histone cluster 2, H2aa3*	**HIST2**	723790	7.5
33	*HLA-G histocompatibility antigen, class I, G*	**HLA-G**	3135	7.2
34	*hydroxysteroid (11-beta) dehydrogenase 1*	**HSD11B1**	3290	7.2
35	*chemokine (C-X-C motif) receptor 7*	**CXCR7**	57007	7.0
36	*major histocompatibility complex, class I, B*	**HLA-B**	3106	6.3
37	*nuclear receptor coactivator 7*	**NCOA7**	135112	6.1
38	*myxovirus (influenza virus) resistance 2 (mouse)*	**MX2**	4600	5.8
39	*radical S-adenosyl methionine domain containing 2*	**RSAD2**	91543	5.6
40	*TNFAIP3 interacting protein 1*	**TNIP1**	10318	5.5
41	*tissue factor pathway inhibitor 2*	**TFPI2**	7980	5.4
42	*interleukin 32*	**IL32**	9235	5.3
43	*Nibrin*	**NBN**	4683	5.2
44	*receptor (chemosensory) transporter protein 4*	**RTP4**	64108	5.2
45	*baculoviral IAP repeat-containing 3*	**BIRC3**	330	5.1
46	*interleukin 7 receptor /// interleukin 7 receptor*	**IL7R**	3575	5.0
47	*transporter 1, ATP-binding cassette, sub-family B (MDR/TAP)*	**TAP1**	6890	5.0
48	*papilin, proteoglycan-like sulfated glycoprotein*	**PAPLN**	89932	4.9
49	*Major histocompatibility complex, class I, F*	**HLA-F**	3134	4.9
50	*myosin, light chain kinase*	**MYLK**	4638	4.9

**Down-regulated genes**				

1	*extracellular link domain containing 1*	**XLKD1**	10894	11.1
2	*chemokine (C-C motif) ligand 14*	**CCL14**	6358	11.0
3	*ADAM metallopeptidase with thrombospondin type 1 motif, 18*	**ADAMTS18**	170692	7.8
4	*LIM domain binding 2*	**LDB2**	9079	6.7
5	*keratin 18*	**KRT18**	3875	5.3
6	*dual specificity phosphatase 4*	**DUSP4**	1846	5.3
7	*ribonuclease, RNase A family, 1 (pancreatic)*	**RNASE1**	6035	4.7
8	*periostin, osteoblast specific factor*	**POSTN**	10631	4.7
9	*insulin-like growth factor binding protein 5*	**IGFBP5**	3488	4.6
10	*regulator of G-protein signalling 5*	**RGS5**	8490	4.6
11	*uroplakin 1B /// regulator of G-protein signalling 5*	**UPK1B**	7348	4.5
12	*regulator of G-protein signalling 4*	**RGS4**	5999	5.5
13	*Palmdelphin*	**PALMD**	54873	3.8
14	*latent transforming growth factor beta binding protein 2*	**LTBP2**	4053	3.2
15	C-X-C chemokine receptor type 4	**CXCR4**	7852	3.1

### Identification of K13-modulated biologically relevant networks

In order to find biological interactions among these 174 genes, pathway analysis was carried out by the Ingenuity Pathway Analysis (IPA) software tool. It was found that 156 of the 174 genes mapped to genetic networks as defined by the IPA tool (Additional File 3). These networks are based on known functional interactions between the gene products as described in the literature. The tool then associates these networks with known biological pathways. Eight networks were affected significantly by K13 expression as they had more of the identified genes present than would be expected by chance (score of >20). These networks were associated with important cancer-related cellular events such as cell death, cellular growth and proliferation, cellular movement, immune response, inflammatory diseases, immune responses and hematological diseases (Additional File 3). The pathway (#1), which is associated with cell death, inflammatory diseases and immunological diseases, was identified as the most significantly influenced by K13. This pathway contained 27 genes with a highly significant score of 51. Consistent with the known ability of K13 to activate the NF-κB pathway, the IPA identified the NF-κB pathway as a key pathway linked to the gene networks perturbed by K13 activity in HUVECs (Additional File 4). Additionally, pathway analysis by Gene Spring GX10 identified NF-κB as the major pathway linked to K13 activity (p < 0.05).

We next performed Gene Ontology (GO) analysis on the microarray data. The GO analysis inquires into the functions of all significantly affected genes regardless of their mutual relationship and differs in this aspect from Pathway Analysis. GO analysis identified 21 categories of genes to be significantly affected in HUVEC-K13-ER^TAM ^cells upon 4OHT treatment (Table 2). The GO categories of immune response, cellular movement, inflammatory disease, hematological system development, cancer and cell-to-cell signaling were the statistically most over-represented, reflecting the predominance of chemokines and cytokines among the K13-induced genes. Thus, both analyses (by Pathway Analysis and by Ontology) were concordant in the finding that cell signaling, immune response and inflammation were the dominant categories among the known affected functions of K13.

**Table 2:** Gene Ontology of vFLIP K13 affected genes.

**S. No.**	**Molecular Function and Disease**	**p-value**	**Genes(n)**
1	Immune Response	5.18E-22 - 1.76E-05	63
2	Cellular Movement	8.19E-19 - 1.83E-05	50
3	Inflammatory Disease	8.38E-16 - 1.22E-05	37
4	Hematological System Development and Function	8.94E-16 - 1.83E-05	53
5	Cancer	2.64E-15 - 1.83E-05	73
6	Cell-To-Cell Signaling and Interaction	3.71E-15 - 1.83E-05	47
7	Tissue Morphology	2.98E-14 - 9.68E-06	33
8	Immune and Lymphatic System Development and Function	2.68E-13 - 1.76E-05	44
9	Connective Tissues Disorders	3.99E-13 - 7.43E-06	21
10	Skeletal and Muscular Disorders	3.99E-13 - 7.43E-06	24
11	Cellular Growth and Proliferation	1.64E-12 - 1.45E-05	73
12	Cell death	1.27E-11 - 1.87E-05	52
13	Immunological Disease	2.47E-09 - 1.87E-05	32
14	Tissue Development	4.14E-11 - 1.76E-05	51
15	Cell Signaling	1.73E-10 - 7.56E-06	71
16	Cellular Development	3.41E-10 - 1.70E-05	32
17	Hematological Disease	4.29E-10 - 1.79E-05	39
18	Neurological Disease	2.34E-09 - 1.83E-05	8
19	Cell Cycle	4.54E-09 - 1.44E-05	7
20	Respiratory Disease	3.20E-08 - 1.61E-06	14
21	Viral Function	3.29E-08 - 3.29E-08	11

### Confirmation of microarray results by a multiplex cytokine assay (Luminex) and comparative analysis of chemokines and cytokines induced by K13 and KSHV

The microarray data analysis suggested that expression of a number of chemokines and cytokines genes was highly induced following treatment of K13-ER^TAM^-expressing HUVECs with 4OHT. In order to determine whether the changes in the mRNA level results in a parallel increase in the secretion of the cytokines and chemokines in the cellular supernatants, we used a multiplex cytokine assay to examine the secretion of cytokines and chemokines in the supernatants of vector- and K13-ER^TAM^-expressing HUVECs that had been either mock treated or treated with 4OHT for 48 hours. For comparison, we also included supernatant from HUVECs that have been infected with KSHV for 48 h. We selected the 48 h time point for the comparative analysis as a qRT-PCR analysis revealed that the transient increase in lytic gene expression seen immediately following infection with KSHV returns to near baseline level by this time point while the expression of latent genes (e.g. K13 and LANA) is still maintained (Additional File 5). As shown in Table 3, while 4OHT treatment had no significant effect on the chemokine/cytokine secretion by the control vector-expressing HUVECs, it significantly upregulated the secretion of several chemokines and cytokines in the K13-ER^TAM^-expressing cells, thus confirming the microarray results. The chemokines and cytokines whose expression was strongly (≥10 fold) induced following 4OHT treatment of HUVEC-K13-ER^TAM ^included IL-6 (10 fold), GM-CSF/CSF2 (193 fold), MCP-1 (155 fold), CCL5/RANTES (36 fold), G-CSF/CSF3 (59 fold), CXCL10/IP-10 (149 fold) and IL-12p40 (12 fold). Remarkably, all the above chemokines and cytokines, with the sole exception of CCL5, were also induced in the supernatant from HUVECs that had been infected with KSHV for 48 h. The cytokines/chemokines with modest (2 to 9 fold) induction following 4OHT treatment of K13-ER^TAM ^HUVECs included IL-8, TNFα, Eotaxin, MIP1α, IL-15, IFNα, MIG, IL-7, GROα, and MCP-3. Again, all these cytokines/chemokines, with the exception of TNFα and IL-15, showed more than 2 fold induction following KSHV infection. Taken collectively, the above results provide further validation of our microarray analysis and suggest that K13 plays a major role in the upregulation of chemokines and cytokines expression following KSHV infection.

**Table 3:** Luminex-based multiplex cytokine assay showing the expression of chemokines and cytokines in the supernatants of HUVEC Vector and K13-ER cells with and without 4OHT treatment and following infection with KSHV.

**Cytokines**	**Vector Mock treated**	**Vector + 4OHT**	**K13-ER-Mock treated**	**K13-ER+4OHT**	**KSHV Virus**	**Fold change K13-ER**	**Fold change KSHV**	**Fold change based on Microarray data**
**IL-1b**	155.5	193.5	148	200	200	1.3	1.3	1.07
**IL-2**	20	23	20	22	21	1.1	1.05	1.01
**IL-4**	15	15	16	23	17	1.4	1.06	-2.9
**IL-5**	5	5	4	7	5	1.75	1.25	1.2
**IL-6**	1946.5	2038	1043.5	10910	11242	10.45	10.77	21.17
**IL-8**	6115	6682	4459.5	10336	10094.5	2.31	2.26	31.5
**IL-10**	1023	985	1003	1011.5	1038.5	1.00	1.03	3.3
**IFN-γ**	24.5	20	21	31.5	27	1.5	1.28	1.9
**CSF2**	13	15	10	1938	51	193.80	5.10	15.1
**TNF-α**	8	10	9	24	14	2.66	1.55	5.6
**EOTAXIN**	5	6	4	10	6	2.50	1.50	3.4
**MCP-1**	3217	2928	85	13178	10235.5	155.03	120.41	71.4
**MIP-1α**	37.5	31	24	117	105	4.87	4.37	3.6
**MIP-1β**	38	43	42.5	65	58	1.52	1.36	1.4
**CCL5**	327	345.5	305.5	11126.5	197	36.42	0.644	48.3
**EGF**	8798	8695	8711	8962	8765	1.02	1.00	16.5
**VEGF**	421	491	466.5	479	447	1.02	0.95	2.3
**FGF-β**	415	428	515	615.5	500	1.19	0.97	10.7
**G-CSF**	95	104	36	2125	604	59.02	16.77	3.9
**HGF**	134.5	143.5	148.5	155.5	264	1.04	1.77	3.2
**CXCL10**	498.5	485.5	70	10453	2259	149.32	32.27	47.7
**IL-12p40**	21	18.5	16.5	197	34	11.93	2.06	97
**IL-13**	7	8	7	13	8	1.85	1.14	1.4
**IL-15**	11	12	10	39	12	3.90	1.20	7.8
**IL-17**	118	139	130	129	132	0.99	1.01	-1.3
**IFN-α**	56	60	40	131	98	3.27	2.45	1.4
**MIG**	13	12	14	39	37.5	2.78	2.67	3.8
**IL-1α**	454.5	521.5	469	752	494.5	1.60	1.05	2.3
**IL-7**	58	50	47	197	181.5	4.19	3.86	2.1
**IL-1Rα**	30	31	28.5	90	59.5	3.15	2.08	3.8
**IL-2R**	12	16	15	21.5	18	1.43	1.20	2.6
**GROα**	13	13	14	110	17	7.85	1.21	7.7
**MCP-2**	26.5	25	23	37	23	1.60	1.00	4.4
**MCP-3**	12	14	10	23	372	2.30	37.20	2.3

### K13 stimulates the promoter of CXCL10/IP-10 via NF-κB activation

**Figure 3:**
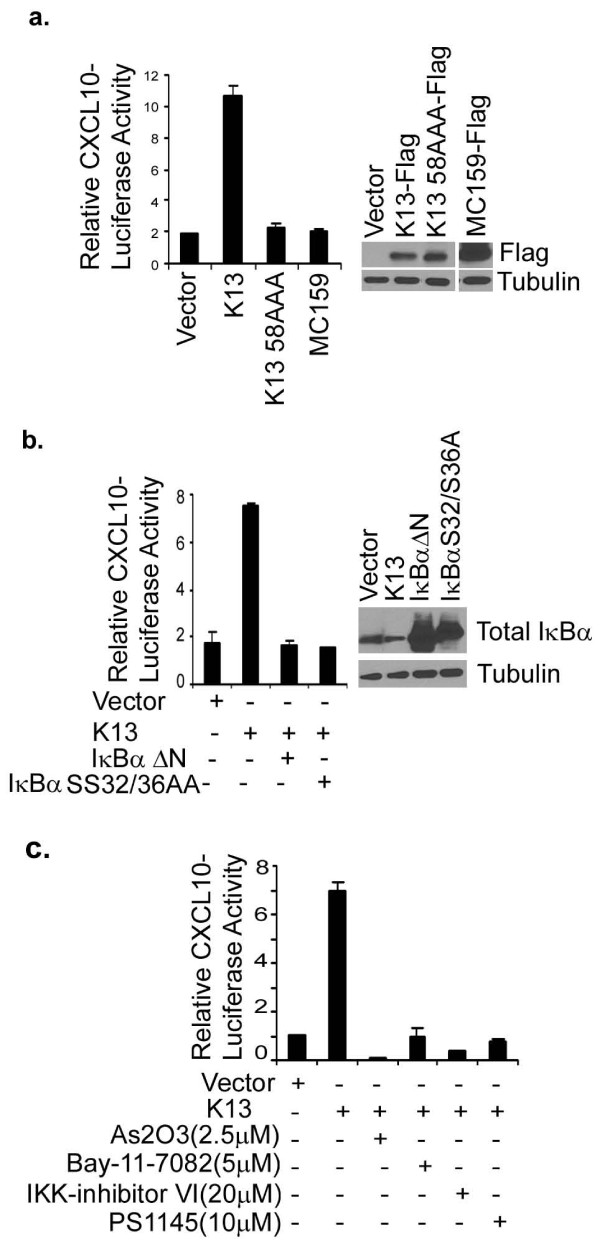
**K13-induced NF-κB activity is critical for the activation of *CXCL10*promoter**. **(a)**. *Right panel*: 293T cells were transfected with an empty vector, wild-type K13, K13-58AAA or MC159 (250 ng/well) along with a *CXCL10 *promoter-driven luciferase constructs (75 ng/well) and a pRSV/LacZ (β-galactosidase) reporter construct (75 ng/well), and the reporter assay performed as described under the Materials and Methods section. *Left panel*: Expression of the transfected proteins in the cell lysates is shown by immunoblotting with an antibody against the Flag epitope tag. Tubulin blot shows equal protein loading. **(b)**. *Right panel*: Dominant-negative mutants of IκBα (IκBαΔN and IκBαSS32/36AA) block K13-induced CXCL10 promoter activity. 293T cells were transfected either with the indicated plasmids and reporter assay performed as described for Figure 3a. The amount of IκBα mutant plasmids (500 ng/well) was five times the amount of vector or K13 (100 ng/well) plasmid and the total amount of transfected DNA was kept constant by adding empty vector. *Left panel*: Expression of the transfected dominant negative mutants of IκBα in the cell lysates is shown by immunoblotting with an antibody against IκBα. Tubulin blot shows equal protein loading. **(c)**. 293T cells were transfected with an empty vector or a vector encoding K13 and subsequently treated with DMSO (vehicle) or the indicated compounds for 16 hours prior to cell lysis. Reporter assay was performed as described for Figure 3a.

To study the mechanism by which K13 upregulates the expression of chemokines, we selected CXCL10 as a representative example. CXCL10 is a powerful chemoattractant for T cells, monocytes/macrophages, NK cells and dendritic cells, and may contribute to the infiltration by inflammatory cells in the KS lesions [24]. To investigate the mechanism by which K13 upregulates CXCL10 expression, human embryonic kidney 293T cells were transfected with a luciferase-based reporter construct containing the CXCL10 gene promoter. While co-expression of K13 strongly activated the CXCL10 promoters, vFLIP MC159 from the molluscum contagiosum virus, which resemble K13 in structure but lacks the ability to activate the NF-κB pathway [10,25], failed to do so (Figure 3a). The involvement of the NF-κB pathway in K13-induced CXCL10 transcriptional activation was confirmed by using an NF-κB-defective mutant of K13, K13-58AAA [14], which failed to activate the CXCL10 promoter (Figure 3a). Furthermore, K13-induced CXCL10 promoter activity was effectively blocked by two phosphorylation-resistant mutants of IκBα (IκBα SS32/36AA and IκBαΔN) (Figure 3b) that are known to block the NF-κB pathway [10], and by treatment with chemical inhibitors of the NF-κB pathway, including Bay-11-7082 [21], IKK inhibitor VI [26], PS1145 [27] and arsenic trioxide [28] (Figure 3c). Collectively, these results support the involvement of the NF-κB pathway in K13-induced upregulation of CXCL10.

### Differential modulation of lymphatic differentiation genes by K13 and KSHV

**Figure 4:**
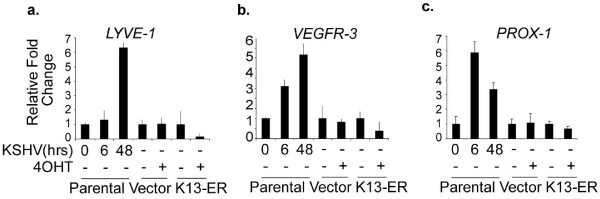
**KSHV infection upregulates the expression of lymphatic markers but K13 fails to do so**. HUVECs were infected with KSHV for the indicated time intervals and the expression of lymphatic markers LYVE-1, VEGFR-3 and PROX-1 was determined by qRT-PCR analysis. In parallel, the expression of the lymphatic markers was determined in HUVECs-K13-ER^TAM^and -vector cells that had been left untreated or treated with 4OHT (50 nM) for 48 h. The experiment was performed as described in the Materials and Methods section.

Kaposi sarcoma spindle cells, the neoplastic cells of the lesion, express markers of lymphatic endothelium [29,30]. KSHV infection of blood vascular endothelial cells not only makes them acquire a spindle shaped phenotype but is also known to induce their differentiation into lymphatic endothelial cells which is accompanied by expression of PROX-1, a master regulator of lymphatic development, and expression of lymphatic markers such as VEGFR-3, Podoplanin and XLKD1/LYVE1 [7-9]. Since ectopic expression of K13 in vascular endothelial cells induces spindle cell transformation [21,22], we examined whether K13 can also mimic the effect of KSHV infection on lymphatic differentiation and induce the expression of lymphatic markers in HUVECs. However, we found that K13 down-regulated the expression of the lymphatic marker gene XLKD1/LYVE1 by 11 fold and had no major effect on the expression of other lymphatic markers, such as PROX1, VEGFR-3, Podoplanin, and CD206 (Table 1). We also used qRT-PCR to confirm the results of microarray analysis. Consistent with the published results, infection of HUVECs with KSHV led to a significant increase in the expression of XLKD1/LYVE1, VEGFR-3 and PROX-1, while treatment of HUVECs-K13-ER^TAM ^with 4OHT failed to do so (Figure 4). Thus, K13 expression is not sufficient to account for the induction of lymphatic differentiation markers in HUVECs observed following KSHV infection.

## Discussion

K13 is one of the few KSHV-encoded latent proteins and is consistently expressed in the KS spindle cells, the hallmark of KS lesions [31]. Ectopic expression of K13 in human vascular endothelial cells is sufficient to make them acquire a spindle-shaped morphology that is associated with NF-κB activation and increased production of a number of genes known to be upregulated in KSHV-infected cells [21,22]. In this study, we provide a comprehensive picture of global transcriptional changes induced by K13 in HUVECs. Our results provide the starting point for future detailed analysis of the K13-induced genes in the pathogenesis of KS.

As K13-induced spindle cell transformation of HUVECs is accompanied by their loss of proliferating potential, it is not possible to generate stable cultures of long-term proliferating HUVECs expressing K13 for the purpose of global gene expression analysis. To circumvent this problem, we took advantage of our previously characterized HUVEC-K13-ER^TAM ^model system in which the K13 activity is dormant in the absence of 4OHT treatment [21]. The HUVEC-K13-ER^TAM ^maintains their normal cobblestone appearance and are indistinguishable from the control vector-expressing cells in their appearance and growth characteristics in the absence of 4OHT treatment. The lack of leakiness in this system was further confirmed by our microarray and qRT-PCR analysis, which revealed no significant difference in the gene expression profile between the vector and K13-ER^TAM^-expressing HUVECs in the absence of 4OHT. While 4OHT treatment significantly changed the expression of a number of genes in K13-ER^TAM ^cells, it had no major effect on gene expression in the control vector cells, thereby confirming that that 4OHT treatment had no major effect on gene expression on its own. Another advantage of the use of this inducible model system is that allowed us to compare the effect of K13 activity on gene expression in the same cell population, thereby avoiding any artifacts due to cell-to-cell (clonal) variation.

It has been proposed that KSHV infection plays a major role in the recruitment of inflammatory cells to the KS lesions by upregulating the expression of chemotactic chemokines and, consistent with this notion, latent infection of vascular endothelial cells with KSHV has been known to upregulate the expression of several cellular chemokines, such as IL-8, GRO-1, MCP-1, NAP-2, CCL5/RANTES and CXCL16 [32,15,21,18,33,22]. The chemokines induced by KSHV infection, such as IL-8, have been also postulated to contribute to neoangiogenesis characteristic of KS lesions. Our analysis revealed that chemokines were the most upregulated genes upon induction of K13 activity in HUVECs, which is consistent with the previous studies [21,22] and two recent studies that were published while this manuscript was under review [34,35]. Notable chemokines genes whose expression was increased significantly by K13 included CCL2 (71 fold), CCL20 (53 fold), CCL5/RANTES (48 fold), CXCL10 (47 fold), CCL3 (33 fold), IL8 (31 fold), CX3CL1 (22 fold), CXCL1 (7) and CXCL5 (13 fold). In addition, expression of genes encoding a number of cytokines (e.g. IL1a, IL6, IL15, IL32, CSF1, CSF2, CSF3 and EBI3) and TNF family ligands (TNFSF13B and Lymphotoxin beta) was significantly increased upon induction of K13 activity. Since chemokines and cytokines collectively represented the most upregulated genes in K13 expressing HUVECs, we confirmed their increased secretion in the supernatant of 4OHT treated K13-ER^TAM ^cells. More importantly, a comparison with KSHV-infected cells revealed that ectopic expression of K13 is sufficient to induce most chemokines/cytokines that are induced by KSHV infection. Since K13 is one of the few KSHV genes that are expressed in latently-infected KS spindle cells, the above results support the hypothesis that K13, either alone or in combination with other viral genes, plays a key role in the up-regulation of chemokines and cytokines and subsequent recruitment of inflammatory cells to the KS lesions.

We observed significant upregulation of adhesion molecules (e.g. ICAM-1, VCAM-1 and E-Selectin), MHC-I, TAP1, TAP2 and tapasin upon induction of K13 activity, which is consistent with recent reports [22,36]. Increased expression of adhesion molecules on vascular endothelial cells expression by K13 might synergize with chemokines to promote the recruitment of inflammatory and blood cells into KS lesions. On the other hand, increased MHC-I expression has been postulated to ensure controlled viral dissemination during latency by promoting cytotoxic T lymphocyte (CTL) proliferation [36]. K13-induced upregulation of adhesion molecules and MHC-1 molecules during natural infection with KSHV may be modulated by concomitant expression of viral lytic proteins, such as the K5 gene product and vIRF1, which have been shown to down-regulate the expression ICAM-1 and MHC-I molecules [36,37].

Infection with KSHV has been reported to contribute to neoangiogenesis, another characteristic feature of KS lesions, by upregulating the expression of several genes involved in the control of vascular modeling and angiogenesis, such as VEGF-A, VEGF-C, angiopoietin-related protein 4, thrombomodulin, and matrix metalloproteinase (MMP-1) [32]. The expression of VEGF-A, VEGF-C and angiopoietin-related protein 4 was not significantly increased, and expression of thrombomodulin was decreased 2-fold upon induction of K13 activity. Additionally, the expression of tissue factor pathway inhibitor 2 (TFPI2), a gene which is a negative inhibitor of aberrant angiogenesis associated with tumor development was increased 5-fold by K13 [38]. However, the expression of semaphorin 3C (SEMA3C) and TNF-alpha-induced protein 2 (TNFAIP2), two invasion and angiogenic factors [39-41], was induced 2 and 21 fold upon activation of K13 activity, respectively. Similarly, the expression of matrix metallopeptidase 10 (MMP10 or Stromolysin 2), an enzyme implicated in the breakdown of extracellular matrix during tumor invasion, metastases and angiogenesis [42], was highly induced (11 fold) upon induction of K13 activity. Finally, K13 activity strongly (21 fold) induced the expression of TNF alpha-induced protein 6 (TNFAIP6, also known as TSG-6), a member of the Link module superfamily that regulates extracellular matrix remodeling and inflammatory response [43]. Thus, K13 activity may contribute to neoangiogenesis in KS lesions via increased production of angiogenic factors, such as IL-8, SEMA3C and TNFAIP2, and to invasion and metastases by stimulating extracellular matrix remodeling through increased production of MMP10 and TNFAIP6.

K13 strongly induced two stress response genes, cyclooxygenase-2 (COX-2) and manganese superoxide dismutase (SOD2), which have been reported previously to be strongly induced by KSHV infection [32]. COX-2 is an angiogenic stress response gene that was recently shown to facilitate latent KSHV gene expression and the establishment and maintenance of latency [44]. SOD2 plays an important role against mitochondrial oxidative stress by diminishing reactive oxygen species [45], and may promote survival of KSHV-infected cells. Furthermore, while this manuscript was under review, an independent study reported upregulation of SOD2 expression by K13 in vascular endothelial cells, which correlated with decreased intracellular superoxide accumulation and increased resistance to superoxide-induced death [34]. Two other anti-apoptotic genes, baculoviral IAP repeat containing 3 (BIRC3/cIAP2) and BCl2-related protein A1 (BCL2A1), were also strongly upregulated upon induction of K13 activity in our study, and may contribute to the survival of KSHV-infected cells.

The genes belonging to the interferon response pathway represented another class of genes whose expression was upregulated upon induction of K13 activity. Notable genes in this class included interferon γ-inducible protein 30 (IFI30), 28 kDa interferon responsive protein (IFRG28), interferon stimulated exonuclease gene 20 kDa (ISG20), guanylate binding protein 1 (GBP1), interferon induced transmembrane protein 1 (IFITM1), interferon regulatory factor-1, -2 and -7, interferon-induced protein 35 (IFI35), interferon-induced protein with tetratricopeptide repeats 3 (IFIT3), interferon omega 1, interferon induced with helicase C domain 1 (IFIHI), and interferon, beta 1.

K13 is a powerful activator of the NF-κB pathway and others and we have previously reported that K13-induced upregulation of proinflammatory cytokines in vascular endothelium cells is associated with NF-κB activation and can be blocked by genetic and pharmacological inhibitors of this pathway [21,22]. Consistent with the ability of K13 to activate NF-κB, most of the genes induced by K13 in the present study are known targets of the NF-κB pathway [46,47], including NFKBIA (IκBα), which is not only a direct target gene of the NF-κB pathway but also a key negative regulator of this pathway. The involvement of the NF-κB pathway in K13-induced transcriptional activation of chemokine genes was further supported by our studies using the CXCL10 promoters. It needs to be noted, however, that the transcriptional activation of genes is usually complex and it is conceivable that NF-κB pathway cooperates with other signaling pathways in the transcriptional activation of some genes.

Finally, we observed that K13 also down-regulated the expression of several genes in HUVECs. In contrast to the upregulated genes, the down-regulated genes were diverse and did not belong to particular functional class. Nevertheless, these genes are known to act as tumor suppressor (e.g. ADAMTS18 and Periostin) [48,49], apoptosis-inducer (e.g. insulin like growth factor binding protein 5) [50], and regulator of vascular integrity (regulator of G-protein signaling 5) [51], suggesting that their down-regulation by K13 may have a causal role in the pathogenesis of KS lesions.

## Conclusion

Our microarray and multiplex cytokine analyses demonstrate that K13 activity may contribute to the upregulation of a majority of genes induced in HUVECs that are latently infected with KSHV. These results are consistent with the notion that K13, a powerful NF-κB activator, is one of the few KSHV genes that are expressed in latently-infected cells. However, interestingly, K13, by itself, can not mimic the effect of KSHV infection in inducing lymphatic reprogramming of blood vascular endothelial cells. Thus, lymphatic differentiation by KSHV may require supplementation by other viral gene activities.

## Abbreviations

HHV8: Human herpesvirus 8; KS: Kaposi's Sarcoma; KSHV: Kaposi's sarcoma-associated herpesvirus; vFLIP: viral Fas-associated death domain-like IL-1β-converting enzyme inhibitory protein; IκB: Inhibitor of κB; PEL: Primary effusion lymphoma; NF-κB: Nuclear Factor kappa B; 4OHT: 4-hydroxytamoxifen; MSCV: Murine stem cell virus; LANA: Latency-associated nuclear antigen; LYVE1: Lymphatic vessel endothelial hyaluronan receptor 1; PROX1: prospero-related homeobox 1; VEGFR3: Vascular endothelial growth factor receptor 3.

## Competing interests

The authors declare that they have no competing interests.

## Authors' contributions

VP analyzed the microarray and multiplex cytokine array data, carried out qRT-PCR experiments and wrote the draft of the manuscript. HM performed immunoblotting and CXCL10 reporter assays. SC assisted with reporter assays. PMC conceived and designed the study, and wrote the final draft of the paper. All authors read and approved the final manuscript.

## Pre-publication history

The pre-publication history for this paper can be accessed here:



## Supplementary Material

Additional file 1 **List of primers used in real-time PCR**. Click here for file

Additional file 2 **List of differentially affected genes in 4OHT treated K13-ER^TAM ^HUVECs**. List of the upregulated and downregulated genes by K13 using a 2-fold threshold change. Click here for file

Additional file 3 **Genetic networks affected by K13-ER^TAM ^in HUVECs**. Bold genes are those identified by the microarray analysis. Other genes were either not on the expression array or not significantly affected. Arrows indicate up (↑) or down (↓) regulation of the gene by K13. A score of >20 was considered significant (p < 0.001). Click here for file

Additional file 4 **Ingenuity Network analysis showing interactions between K13-responsive genes**. The network as determined by IPA is shown graphically as nodes (symbols representing genes) and lines/arrows (biological relationship between genes). The degree of differential expression is shown beneath the name of the gene symbol. Lines and arrows displayed with various labels that describe specific relationship between nodes. These include: I, inhibition; L, proteolysis; P, phosphorylation: T, transcription. The absence of label indicates binding only. Dotted lines indicate indirect interaction while direct interaction is indicated by solid lines. Further detailed explanation of various nodes and there relationship symbols can be found at Ingenuity pathway analysis web page . Click here for file

Additional file 5 **Time-course of latent and lytic genes induction following KSHV infection**. HUVECs were infected with KHSV for the indicated time intervals and induction of latent (K13 and LANA) and lytic (ORFK8.1 and vIL6) genes determined by qRT-PCR. Click here for file
