# Supplementary material for: Integrated microarray and multiplex cytokine analyses of Kaposi's Sarcoma Associated Herpesvirus viral FLICE Inhibitory Protein K13 affected genes and cytokines in human blood vascular endothelial cells
Source: BMC Med Genomics. 2009 Aug 6;2:50. doi: 10.1186/1755-8794-2-50 (PMC2732924; doi:10.1186/1755-8794-2-50)
Supplement: Additional file 1 — List of primers used in real-time PCR. [file 1755-8794-2-50-S1.pdf]

Supplementary Table1. Primers used in the real time PCR.

VCAM1 Forward	GGGACCACATCTACGCTGACA
VCAM1 Reverse	CCTGTCTGCATCCTCCAGAAA
CSF2 Forward	GGCCAGCCACTACAAGCAGCACT
CSF2 Reverse	CAAAGGGGATGACAAGCAGAAAG
SOD2 Forward	CGACCTGCCCTACGACTACG
SOD2 Reverse	TGACCACCACCATTGAACTT
CXCL3 Forward	AAGAAGCTTATCAGCGTATCAT
CXCL3 Reverse	AATAAGTAGAACCCTCGTAAGAAA
IGFBP5 Forward	GAGCTGAAGGCTGAAGCAGT
IGFBP5 Reverse	GAATCCTTTGCGGTCACAAT
BIRC3 Forward	ACTTGAACAGCTGCTATCCACATC
BIRC3 Reverse	GTTGCTAGGATTTTTCTCTGAACTGTC
COX2 Forward	TGAGCATCTACGGTTTGCTG
COX2 Reverse	AACTGCTCATCACCCCATTC
IL8 Forward	GCCAACACAGAAATTATTGTAAAGCTT
IL8 Reverse	CCTCTGCACCCAGTTTTCCTT
CCL5 Forward	CCCAGCAGTCGTCTTTGTCA
CCL5 Reverse	TCCCGAACCCATTTCTTCTCT
CXCL10 Forward	GAGCCTACAGCAGAGGAACC
CXCL10 Reverse	GAGTCAGAAAGATAAGGCAGC
IL6 Forward	GGTACATCCTCGACGGCATCT
IL6 Reverse	GTGCCTCTTTGCTGCTTTCAC
K13Fowrad	GGATGCCCTAATGTCAATGC
K13 Reverse	GGCGATAGTGTTGGAGTGT
ORF50 Forward	CACAAAAATGGCGCAAGATGA
ORF50 Reverse	TGGTAGAGTTGGGCCTTCAGTT
ORFK8.1 Forward	AAAGCGTCCAGGCCACCACAGA
ORFK8.1Reverse	GGCAGAAAATGGCACACGGTTAC
VEGFR-3 Forward	CTGCTGGAGGAAAAGTCTGG
VEGFR-3 reverse	GTCTTGATGTCTGCGTGGG
PROX-1 Forward	TTGACATTGGAGTGAAAAGGACG
PROX-1 reverse	TGCTCAGAACCTTGGGGATTC
LYVE-1 Forward	TCACTTCCATCTGGACTACGAG
LYVE-1 Reverse	CACAAGGGTGATCCCCATAATTC
