# Supplementary material for: Integrated microarray and multiplex cytokine analyses of Kaposi's Sarcoma Associated Herpesvirus viral FLICE Inhibitory Protein K13 affected genes and cytokines in human blood vascular endothelial cells
Source: BMC Med Genomics. 2009 Aug 6;2:50. doi: 10.1186/1755-8794-2-50 (PMC2732924; doi:10.1186/1755-8794-2-50)
Supplement: Additional file 2 — List of differentially affected genes in 4OHT treated K13-ERTAM HUVECs. List of the upregulated and downregulated genes by K13 using a 2-fold threshold change. [file 1755-8794-2-50-S2.pdf]

Supplementary Table 2: Summary of differentially regulated gene in 4-OHT treated K13 ER-transduced HUVEC.

S.No.	Gene Name	Gene	Entrez Gene	Fold Change
**Up-regulated genes**				
1	*chemokine (C-C motif) ligand 2*	**CCL2**	6347	71.4
2	*ubiquitin D*	**UBD**	10537	58.4
3	*chemokine (C-C motif) ligand 20*	**CCL20**	6364	53.1
4	*chemokine (C-C motif) ligand 5*	**CCL5**	6352	48.3
5	*chemokine (C-X-C motif) ligand 10*	**CXCL10**	3627	47.7
6	*vascular cell adhesion molecule 1*	**VCAM1**	7412	46.0
7	*selectin E (endothelial adhesion molecule 1)*	**SELE**	6401	35.4
8	*superoxide dismutase 2, mitochondrial*	**SOD2**	6648	35.2
9	*chemokine (C-X-C motif) ligand 3*	**CXCL3**	2921	33.8
10	*chemokine (C-X-C motif) ligand 2*	**CXCL2**	2920	32.2
11	*interleukin 8*	**IL8**	3576	31.5
12	*chromosome 15 open reading frame 48*	**C15orf48**	84419	29.6
13	*solute carrier family 7 (cationic amino acid transporter)*	**SLC7A2**	6542	23.2
14	*chemokine (C-X3-C motif) ligand 1*	**CX3CL1**	6376	22.3
15	*intercellular adhesion molecule 1 (CD54)*	**ICAM1**	3383	22.2
16	*tumor necrosis factor, alpha-induced protein 6*	**TNFAIP6**	7130	21.2
17	*interleukin 6 (interferon, beta 2)*	**IL6**	3569	21.1
18	*colony stimulating factor 2 (granulocyte-macrophage)*	**CSF2**	1437	15.1
19	*interferon stimulated exonuclease gene 20kDa*	**ISG20**	3669	13.6
20	*chemokine (C-X-C motif) ligand 5*	**CXCL5**	6374	13.0
21	*laminin, gamma 2*	**LAMC2**	3918	12.7
22	*tumor necrosis factor, alpha-induced protein 3*	**TNFAIP3**	7128	12.5
23	*matrix metallopeptidase 10 (stromelysin 2)*	**MMP10**	4319	11.5
24	*tumor necrosis factor, alpha-induced protein 2*	**TNFAIP2**	7127	11.3
25	*Epstein-Barr virus induced gene 3*	**EBI3**	10148	9.8
26	*tumor necrosis factor (ligand) superfamily, member 13b*	**TNFSF13B**	10673	9.6
27	*phospholipase A1 member A*	**PLA1A**	51365	9.4
28	*GTP cyclohydrolase 1 (dopa-responsive dystonia)*	**GCH1**	2643	9.0
29	*nuclear factor of kappa light polypeptide gene enhancer, alpha*	**NFKBIA**	4792	8.3
30	*proteasome (prosome, macropain) subunit, beta type, 9*	**PSMB9**	5698	7.9
31	*Chemokine (C-X-C motif) 1*	**CXCl1**	2919	7.7
32	*histone cluster 2, H2aa3*	**HIST2**	723790	7.5
33	*HLA-G histocompatibility antigen, class I, G*	**HLA-G**	3135	7.2
34	*hydroxysteroid (11-beta) dehydrogenase 1*	**HSD11B1**	3290	7.2
35	*chemokine (C-X-C motif) receptor 7*	**CXCR7**	57007	7.0
36	*major histocompatibility complex, class I, B*	**HLA-B**	3106	6.3
37	*nuclear receptor coactivator 7*	**NCOA7**	135112	6.1
38	*myxovirus (influenza virus) resistance 2 (mouse)*	**MX2**	4600	5.8
39	*radical S-adenosyl methionine domain containing 2*	**RSAD2**	91543	5.6
40	*TNFAIP3 interacting protein 1*	**TNIP1**	10318	5.5
41	*tissue factor pathway inhibitor 2*	**TFPI2**	7980	5.4
42	*interleukin 32*	**IL32**	9235	5.3
43	*nibrin*	**NBN**	4683	5.2
44	*receptor (chemosensory) transporter protein 4*	**RTP4**	64108	5.2
45	*baculoviral IAP repeat-containing 3*	**BIRC3**	330	5.1
46	*interleukin 7 receptor /// interleukin 7 receptor*	**IL7R**	3575	5.0
47	*transporter 1, ATP-binding cassette, sub-family B (MDR/TAP)*	**TAP1**	6890	5.0
48	*papilin, proteoglycan-like sulfated glycoprotein*	**PAPLN**	89932	4.9
49	*Major histocompatibility complex, class I, F*	**HLA-F**	3134	4.9
50	*myosin, light chain kinase*	**MYLK**	4638	4.9
51	*interferon induced transmembrane protein 1 (9-27)*	**IFITM1**	8519	4.8
52	*interferon-induced protein 35*	**IFI35**	3430	4.8
53	*proline-serine-threonine phosphatase interacting protein 2*	**PSTPIP2**	9050	4.8
54	*bone marrow stromal cell antigen 2*	**BST2**	684	4.7
55	*2'-5'-oligoadenylate synthetase 3, 100kDa*	**OAS3**	4940	4.7
56	*caspase 1, apoptosis-related cysteine peptidase (interleukin 1, beta, convertase)*	**CASP1**	834	4.6
57	*interferon-induced protein with tetratricopeptide repeats 3*	**IFIT3**	3437	4.5
58	*CNKSR family member 3*	**CNKSR3**	154043	4.5
59	*likely ortholog of mouse lung-inducible Neutralized-related C3HC4 RING domain*	**LINCR**	93082	4.5
60	*Duffy blood group, chemokine receptor*	**DARC**	2532	4.4
61	*butyrophilin, subfamily 3, member A1*	**BTN3A1**	11119	4.3
62	*solute carrier family 2 (facilitated glucose transporter), member 6*	**SLC2A6**	11182	4.3
63	*lipase, endothelial*	**LIPG**	9388	4.3

64	*guanylate binding protein 1, interferon-inducible, 67kDa*	**GBP1**	2633	4.2
65	*prolactin receptor*	**PRLR**	5618	4.1
66	*cytochrome b5 type A (microsomal)*	**CYB5A**	1528	4.1
67	*CD69 molecule*	**CD69**	969	4.1
68	*colony stimulating factor 3 (granulocyte)*	**CSF3**	1440	3.9
69	*C-type lectin domain family 4, member E*	**CLEC4E**	26253	3.8
70	*laminin, beta 3*	**LAMB3**	3914	3.8
71	*kynureninase (L-kynurenine hydrolase)*	**KYNU**	8942	3.7
72	*RAD50 homolog (S. cerevisiae)*	**RAD50**	10111	3.6
73	*cathepsin H*	**CTSH**	1512	3.5
74	*G protein-coupled receptor, family C, group 5, member B*	**GPRC5B**	51704	3.5
75	*inhibin, beta A (activin A, activin AB alpha polypeptide)*	**INHBA**	3624	3.5
76	*cathepsin S*	**CTSS**	1520	3.5
77	*colony stimulating factor 1 (macrophage)*	**CSF1**	1435	3.5
78	*cathepsin K*	**CTSK**	1513	3.5
79	*chromosome 11 open reading frame 17*	**C11orf17**	56672	3.4
80	*purinergic receptor P2X, ligand-gated ion channel, 4*	**P2RX4**	5025	3.3
81	*indoleamine-pyrrole 2,3 dioxygenase*	**INDO**	3620	3.3
82	*family with sequence similarity 129, member A*	**FAM129A**	116496	3.3
83	*interleukin 18 receptor 1*	**IL18R1**	8809	3.3
84	*phospholipase A2, group IVA (cytosolic, calcium-dependent)*	**PLA2G4A**	5321	3.3
85	*Wilms tumor 1 associated protein*	**WTAP**	9589	3.3
86	*glutamine-fructose-6-phosphate transaminase 2*	**GFPT2**	9945	3.2
87	*complement component 1, r subcomponent*	**C1R**	715	3.2
88	*BCL2-related protein A1*	**BCL2A1**	597	3.2
89	*hyaluronan and proteoglycan link protein 3*	**HAPLN3**	145864	3.2
90	*TAP binding protein (tapasin)*	**TAPBP**	6892	3.2
91	*interleukin-1 receptor-associated kinase 2*	**IRAK2**	3656	3.2
92	*complement factor B*	**CFB**	629	3.1
93	*lysosomal-associated membrane protein 3*	**LAMP3**	27074	3.1
94	*C1q and tumor necrosis factor related protein 1*	**C1QTNF1**	114897	3.0
95	*solute carrier family 25, member 37*	**SLC25A37**	51312	3.0
96	*Prostaglandin-endoperoxide synthase 2*	**PTGS2**	5743	3.0
97	*retinoic acid receptor responder (tazarotene induced) 1*	**RARRES1**	5918	3.0
98	*NK3 transcription factor related, locus 1 (Drosophila)*	**NKX3-1**	4824	2.9
99	*lymphocyte antigen 6 complex, locus E*	**LY6E**	4061	2.9
100	*phospholipase A2, group IVC (cytosolic, calcium-independent)*	**PLA2G4C**	8605	2.8
101	*lipopolysaccharide-induced TNF factor*	**LITAF**	9516	2.7
102	*serpin peptidase inhibitor, clade B (ovalbumin), member 2*	**SERPINB2**	5055	2.7
103	*TNF receptor-associated factor 1*	**TRAF1**	7185	2.7
104	*butyrophilin, subfamily 3, member A3*	**BTN3A3**	10384	2.7
105	*XIAP associated factor-1*	**BIRC4BP**	54739	2.7
106	*transmembrane protein induced by tumor necrosis factor alpha*	**TMPIT**	83862	2.7
107	*complement component 1, s subcomponent*	**C1S**	716	2.6
108	*toll-like receptor 3*	**TLR3**	7098	2.5
109	*zinc finger CCCH-type containing 12C*	**ZC3H12C**	85463	2.5
110	*tumor necrosis factor receptor superfamily, member 9*	**TNFRSF9**	3604	2.5
111	*solute carrier family 15, member 3*	**SLC15A3**	51296	2.5
112	*interleukin 15*	**IL15**	3600	2.4
113	*receptor-interacting serine-threonine kinase 2*	**RIPK2**	8767	2.4
114	*lectin, galactoside-binding, soluble, 3 binding protein*	**LGALS3BP**	3959	2.4
115	*CD38 molecule*	**CD38**	952	2.3
116	*interleukin 1, alpha*	**IL1A**	3552	2.3
117	*lymphotoxin beta (TNF superfamily, member 3)*	**LTB**	4050	2.3
118	*C-type lectin domain family 1, member A*	**CLEC1A**	51267	2.2
119	*likely ortholog of mouse D11lgp2*	**LGP2**	79132	2.2
120	*ADP-ribosyltransferase 4 (Dombrock blood group)*	**ART4**	420	2.2
121	*coagulation factor II (thrombin) receptor-like 1*	**F2RL1**	2150	2.2
122	*guanylate binding protein 4*	**GBP4**	115361	2.1
123	*sema domain, immunoglobulin domain (Ig), short basic domain, secreted, 3C*	**SEMA3C**	10512	2.0
**Down-regultated genes**				
124	*extracellular link domain containing 1*	**XLKD1**	10894	11.1
125	*chemokine (C-C motif) ligand 14*	**CCL14**	6358	11.0
126	*ADAM metallopeptidase with thrombospondin type 1 motif, 18*	**ADAMTS18**	170692	7.8
127	*LIM domain binding 2*	**LDB2**	9079	6.7
128	*keratin 18*	**KRT18**	3875	5.3
129	*dual specificity phosphatase 4*	**DUSP4**	1846	5.3
130	*ribonuclease, RNase A family, 1 (pancreatic)*	**RNASE1**	6035	4.7

131	*periostin, osteoblast specific factor*	**POSTN**	10631	4.7
132	*insulin-like growth factor binding protein 5*	**IGFBP5**	3488	4.6
133	*regulator of G-protein signalling 5*	**RGS5**	8490	4.6
134	*uroplakin 1B (regulator of G-protein signalling 5)*	**UPK1B**	7348	4.5
135	*regulator of G-protein signalling 4*	**RGS4**	5999	4.4
136	*keratin 7*	**KRT7**	3855	4.2
137	*apelin, AGTRL1 ligand*	**APLN**	8862	4.0
138	*furry homolog (Drosophila)*	**FRY**	10129	3.9
139	*prickle homolog 1 (Drosophila)*	**PRICKLE1**	144165	3.8
140	*palmdelphin*	**PALMD**	54873	3.8
141	*netrin 4*	**NTN4**	59277	3.7
142	*endomucin*	**EMCN**	51705	3.5
143	*BMX non-receptor tyrosine kinase*	**BMX**	660	3.4
144	*LIM homeobox 6*	**LHX6**	26468	3.3
145	*response gene to complement 32*	**RGC32**	28984	3.3
146	*latent transforming growth factor beta binding protein 2*	**LTBP2**	4053	3.2
147	C-X-C chemokine receptor type 4	**CXCR4**	7852	3.1
148	*hypothetical protein*	**DKFZP686**	22998	2.9
149	*aldehyde dehydrogenase 1 family, member A1*	**ALDH1A1**	216	2.9
150	*thioredoxin reductase 2*	**TXNRD2**	10587	2.8
151	*G protein-coupled receptor 116*	**GPR116**	221395	2.8
152	*carboxymethylenebutenolidase homolog (Pseudomonas)*	**CMBL**	134147	2.7
153	*importin 11*	**IPO11**	51194	2.7
154	*contactin associated protein-like 3*	**CNTNAP3**	389734	2.6
155	*coxsackie virus and adenovirus receptor*	**CXADR**	1525	2.6
156	*anthrax toxin receptor 1*	**ANTXR1**	84168	2.6
157	*integrin, alpha 10*	**ITGA10**	8515	2.5
158	*dysferlin, limb girdle muscular dystrophy 2B (autosomal recessive)*	**DYSF**	8291	2.5
159	*chromosome 10 open reading frame 116*	**C10orf116**	10974	2.5
160	*neuronal growth regulator 1*	**NEGR1**	257194	2.4
161	*T cell receptor beta variable 21-1*	**TRBV21-1**	28566	2.4
162	*homeodomain-only protein*	**HOP**	84525	2.4
163	*CD59 molecule, complement regulatory protein*	**CD59**	966	2.4
164	*sema domain, transmembrane domain (TM), and cytoplasmic domain, 6A*	**SEMA6A**	57556	2.3
165	*integrin, beta 4*	**ITGB4**	3691	2.3
166	*glutathione peroxidase 3 (plasma)*	**GPX3**	2878	2.3
167	*mal, T-cell differentiation protein-like*	**MALL**	7851	2.2
168	*cytochrome P450, family 1, subfamily A, polypeptide 1*	**CYP1A1**	1543	2.2
169	*thrombomodulin*	**THBD**	7056	2.2
170	*interleukin 33*	**IL33**	90865	2.2
171	*DIRAS family, GTP-binding RAS-like 3*	**DIRAS3**	9077	2.1
172	*protein phosphatase 1, regulatory (inhibitor) subunit 16B*	**PPP1R16B**	26051	2.1
173	*chromosome 20 open reading frame 160*	**C20orf160**	140706	2.0
174	*pyruvate dehydrogenase kinase, isozyme 4*	**PDK4**	5166	2.0
