# Supplementary material for: Integrated microarray and multiplex cytokine analyses of Kaposi's Sarcoma Associated Herpesvirus viral FLICE Inhibitory Protein K13 affected genes and cytokines in human blood vascular endothelial cells
Source: BMC Med Genomics. 2009 Aug 6;2:50. doi: 10.1186/1755-8794-2-50 (PMC2732924; doi:10.1186/1755-8794-2-50)
Supplement: Additional file 3 — Genetic networks affected by K13-ERTAM in HUVECs. Bold genes are those identified by the microarray analysis. Other genes were either not on the expression array or not significantly affected. Arrows indicate up (↑) or down (↓) regulation of the gene by K13. A score of >20 was considered significant (p < 0.001). [file 1755-8794-2-50-S3.pdf]

Supplementary Table 3: Genetic networks affected by vFLIP K13 in HUVEC cells

S.No.	Genes in Ingenuity Network	Score	Focus Genes	Associated Pathways
1	↑**BCL2A1,** ↑**BIRC3,** ↑**BIRC4BP,** ↑**CASP1**,Caspase, ↑**CCL2,**Cpla2, ↑**CSF2**, ↑**CSF3,** ↑**F2RL1,** ↑**IFI35,** ↑**IFITM1,**Ige, ↑**IL15,** ↓**IL33,** ↑**IL18R1,** ↑**LTB,**Mek,Mek1/2, ↑**MYLK,** ↑**NFKBIA,**P38 MAPK, ↑**PLA2G4A,** ↑**PLA2G4C,**PLC, ↑**PSMB9,**↓**RGS4,**↑**RIPK2,**STAT5a/b, ↑**TLR3,** ↑**TNFAIP3,** ↑**TNFRSF9,** ↑**TNFSF13B,**↑**TNIP1,** ↑**TRAF1**	51	27	Cell Death, Inflammatory Disease, Immunological Disease
2	C1q, ↑**C1R,** ↑**C1S,** ↑**CCL20,** ↑**CLEC4E,** ↑**CTSS,** ↑**CXCL5,** Cyclooxygenase,↓**DIRAS3,** ↑**EBI3,** ↑**HLA-B,** ↑**HLA-F,** ↑**HLA-G,**↓**IGFBP5,** Ikb, IKK, IL1, ↑**IL6,** ↑**IL1A,** ↑**INDO,** ↑**INHBA,** ↑**LGALS3BP**,MHC Class I,MHC Class II,MHC I-&alpha, Mmp, ↑**MMP10**, NF-kappaB, ↑**SERPINB2**,Tap, ↑**TAP1,** ↑**TAPBP**,TCR, ↑**TFPI2**,Tnf receptor	38	22	Cell Signaling, Immune Response, Cancer
3	Akt,↓**APLN,**↓**BMX**,C8,↑**CCL5**,CD3,Creb, ↑**CSF1,** ↑**CXCL2,** ↑**CXCL3,**↑**CXCL10,** ↑**DARC,**G alphai,Hsp27, ↑**ICAM1,** ↑**IL8,** ↑**IL32,** ↑**IL7R,**Integrin,↑**IRAK2,** Jnk, ↑**NBN**,p70 S6k,Pdgf,PI3K, ↑**PTGS2**,Rac, ↑**RAD50**,Rap1,Ras, ↑**SELE**,Sod,Tgf beta, ↑**VCAM1**,Vegf	29	18	Cell-To-Cell Signaling and Interaction, Hematological System Development and Function, Immune Response
4	ADAM17,ADORA2B,ATPase, ↑**CCL2**,CCL7,CCR1,CCR2, CCR6,CD74,chondroitin sulfate, ↑**CLEC4E**↑**CTSK,** ↑**CTSS,**↑**CX3CL1,**CX3CR1, ↑**CXCL3,**↓**DYSF,** ↑**GPRC5B,** ↑**HAPLN3,**HEXB, ↑**IFITM1,**IFNG,IL15RA, ↑**ISG20,**↓**LDB2,** ↑**LY6E,** ↑**MX2,**↑**RARRES1**,SMARCA4,STAT4,TNFSF11, ↑**UBD**,Ubiquitin,↑**VCAM1,** ↑**ZC3H12C**	29	18	Cell-To-Cell Signaling and Interaction, Hematological System Development and Function, Immune Response
5	ACAN,AFP,ALB,ANPEP,ATF4, ↑**BST2,** ↑**BTN3A3**,C3,C5, CAMP,CD3, ↑**CD38,** ↑**CFB,** ↑**CXCL2,** ↑**CXCL3,** CYP2E1, EREG,↑**GBP1,** ↑**GCH1,** ↑**IFIT3,** ↑**LAMP3,** ↑**LITAF,** LPL,MTTP, ↑**OAS3**,POU2F1, PTX3, ↓**RGS5**, SLC10A1, ↑**SLC7A2**,TCF1,TNF,↑**TNFAIP2,** ↑**TNFAIP6,** ↑**WTAP**	27	17	Inflammatory Disease, Cellular Function and Maintenance,Immune Response
6	AFP,ARG1,BUB1 (includes EG:699),↓**C13ORF15**,Calpain, CAPN2,CCNB2,CD34,CDC2,CDKN3, ↑**CXCL10,** ↑**FAM129A,**↑**GBP4 (includes EG:115361),** IER2, IL9, ↓**IPO11,** ↓**ITGB4**,ITGB7,↑**LAMB3**, LAMB1-2, ↑**LAMC2,** LIMS1,↓**LTBP2**,MYC, ↑**PLA1A**,PLP1,↓**POSTN**, RAN, retinoic acid,↓**RNASE1 (includes EG:6035),** ↑**RSAD2,**TGFB1, ↑**TNFAIP2**,VASP,↓**XLKD1**	23	15	Cellular Movement, Dermatological Diseases and Conditions,Genetic Disorder
7	ADCYAP1,↓**ALDH1A1,**↓**ANTXR1,** ↑**CCL2**,CCR2,CEBPB, cholesterol, ↑**CNKSR3**,CTNNB1,↓**CXADR,** ↑**CXCL2,** F2,HDC,HLA-DQB2, HNF4A,↓**HOP,**HP, ↑**HSD11B1**, hydrogen peroxide,ITGA1, ↑**LIPG,**↓**MEST,** ↑**NCOA7**, ORM2, ↓**PDK4**,PPBP, PRNP,progesterone,PROS1, ↑**PSTPIP2**,PTGER4,SAA2,SLC10A1, ↑**SLC2A6,**↓**TXNRD2**	23	15	Immune Response,Cell-To-Cell Signaling and Interaction,Cellular Movement
8	AFP,betaestradiol,BTG2,↓**C10ORF116**,CAV2,CCNB2,CCNC, ↑**CD69,** ↑**CTSH,** ↑**CYB5A**, dihydrotestosterone, ↓**EMCN,** ↓**GPX3,**HOXA5,HPGD,KLK3, ↑**KYNU,** ↓**LHX6,** ↓**MALL**, MKI67,NEUROG3,NFE2L2, ↑**NKX31**,NQO1,OXTR, ↑**P2RX4**, PHB,PRNP,↑**SEMA3C,** ↑**SLC25A37**, SURB7, TP53, TRG@, ↑**VCAM1**,VCAN	21	14	Cancer, Gene Expression, Tissue Morphology
