# Supplementary figures and images for: Integrated microarray and multiplex cytokine analyses of Kaposi's Sarcoma Associated Herpesvirus viral FLICE Inhibitory Protein K13 affected genes and cytokines in human blood vascular endothelial cells
Source: BMC Med Genomics. 2009 Aug 6;2:50. doi: 10.1186/1755-8794-2-50 (PMC2732924; doi:10.1186/1755-8794-2-50)

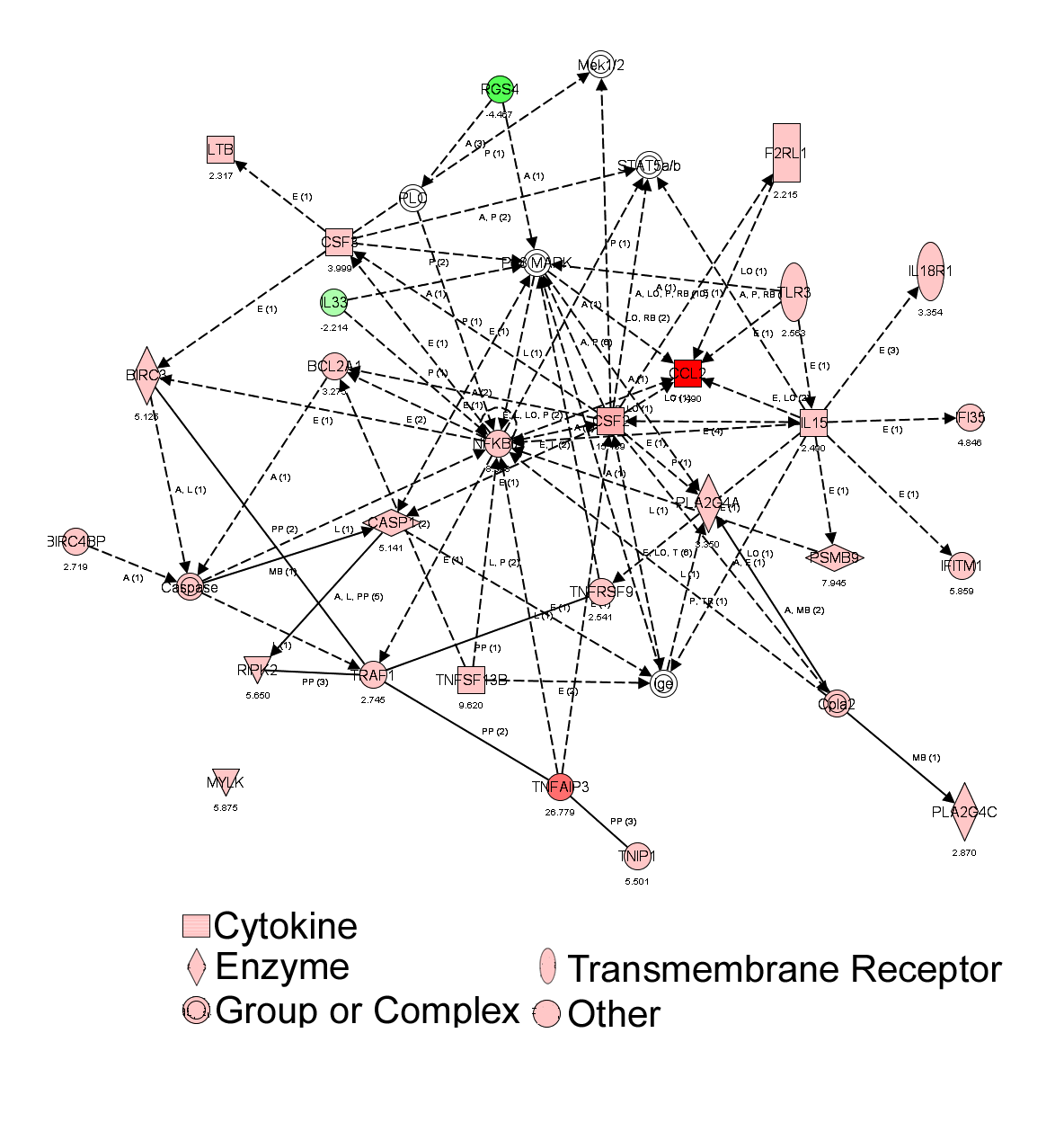

Supplement: Additional file 4 — Ingenuity Network analysis showing interactions between K13-responsive genes. The network as determined by IPA is shown graphically as nodes (symbols representing genes) and lines/arrows (biological relationship between genes). The degree of differential expression is shown beneath the name of the gene symbol. Lines and arrows displayed with various labels that describe specific relationship between nodes. These include: I, inhibition; L, proteolysis; P, phosphorylation: T, transcription. The absence of label indicates binding only. Dotted lines indicate indirect interaction while direct interaction is indicated by solid lines. Further detailed explanation of various nodes and there relationship symbols can be found at Ingenuity pathway analysis web page . [file 1755-8794-2-50-S4.tiff]

**a.**

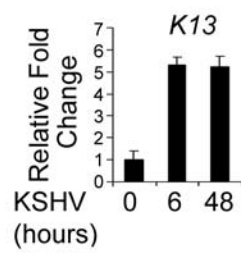

**b.**

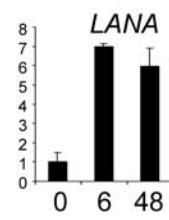

**c.**

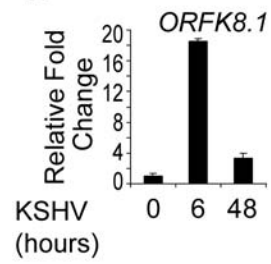

**d.**

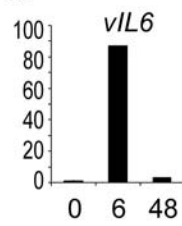

Supplement: Additional file 5 — Time-course of latent and lytic genes induction following KSHV infection. HUVECs were infected with KHSV for the indicated time intervals and induction of latent (K13 and LANA) and lytic (ORFK8.1 and vIL6) genes determined by qRT-PCR. [file 1755-8794-2-50-S5.pdf]
